# Regulatory B cells: the cutting edge of immune tolerance in kidney transplantation

**DOI:** 10.1038/s41419-017-0152-y

**Published:** 2018-01-25

**Authors:** Bo Peng, Yingzi Ming, Cheng Yang

**Affiliations:** 1 Transplantation Center, The Third Xiangya Hospital, Central South University, Changsha Hunan, 410013 P. R. China; 2 Department of Urology, Zhongshan Hospital; Shanghai Key Laboratory of Organ Transplantation, Fudan University, Shanghai, 200032 P. R. China

## Abstract

Kidney transplantation is the optimal treatment for end-stage renal diseases. Although great improvement has been achieved, immune tolerance is still the Holy Grail that every organ transplant practitioner pursues. The role of B cells in transplantation has long been considered simply to serve as precursors of plasma cells, which produce alloantibodies and induce antibody-mediated rejection. Recent research indicates that a specialized subset of B cells plays an important role in immune regulation, which has been well demonstrated in autoimmune diseases, infections, and cancers. This category of regulatory B cells (Bregs) differs from conventional B cells, and they may help develop a novel immunomodulatory therapeutic strategy to achieve immune tolerance in transplantation. Here, we review the latest evidence regarding phenotypes, functions, and effectors of Bregs and discuss their diverse effects on kidney transplantation.

## Facts

- Besides antigen presenting and antibody production, B cells also play a role in immune regulation and tolerance induction through IL-10-dependent and -independent mechanisms.
- A variety of B-cell subsets have been documented as regulatory B cells (Bregs), but no inclusive or specific marker has been found.
- Bregs are usually induced and maintained in response to inflammation, and multiple pathways are involved in different settings.
- Pan-B-cell depletion is not always beneficial for kidney transplantation. It depends on the timing of this approach.
- Operationally tolerant patients of kidney transplantation show a special B-cell signature, and it can be used for prediction of tolerance.

## Open questions

- Are Bregs a distinct lineage of B cells, or just response to inflammation for all of them?
- What exactly is it to make B cells develop into Bregs? Is epigenetics regulation involved?
- What is the relationship between Bregs and other regulatory cells?
- Can Bregs be used for cell therapy to induce tolerance in kidney transplantation?

## Introduction

Kidney transplantation is the optimal treatment for end-stage renal diseases. In past decades, dramatic improvement has been achieved regarding the short-term prognosis of kidney transplantation. However, long-term survival is still not ideal mainly because of chronic rejection (CR) mediated by antibodies^1^. Moreover, lifelong immunosuppressive therapy for most recipients inevitably causes undesired and even severe side effects such as infections, tumors, and metabolic disorders^2^. Therefore, it is the state of immune tolerance that every organ transplant practitioner eagerly desires to achieve.

For many years, T cells remained the focus of research regarding transplantation rejection and tolerance, and the pillar of the current immunosuppressive regimen is T-cell mediated^3^. As for B cells, they have long been considered simply as precursors of plasma cells, which produce alloantibodies and induce antibody-mediated rejection (AMR). However, recent studies highlighted a small population of B cells that showed immune regulatory functions in autoimmune diseases^4^, infections^5^, and cancers^6^, as well as organ transplantation^2,7,8^. This indicates the existence of regulatory B cells (Bregs) that function in more than a detrimental role in transplant immunity. It is time to re-examine the roles of B cells in transplantation and to additionally distinguish regulatory functions from inflammatory functions.

Herein, we review the latest evidence regarding phenotypes, functions, and effectors of Bregs and discuss their diverse effects on kidney transplantation.

## The short history of Bregs

In 1974, B cells were initially presumed to contain a suppressive subset in the model of delayed hypersensitivity in guinea pigs^9^. However, the molecular or biochemical mechanism was unknown, and the conception of “suppressor B cells” was not widely accepted. It was not until the late 1990s that Bregs attracted attention again. Two independent studies showed that autoimmune diseases (experimental autoimmune encephalomyelitis (EAE) and chronic colitis) deteriorated in the B-cell-deficient group, providing further evidence of Bregs and suggesting their function in suppressing inflammation^10,11^. In 2000, Mizoguchi et al. first described B cells that suppressed inflammatory bowel disease using the term “regulatory B cell”^12^. Since then, various subsets of B cells have been shown to regulate immune responses in different settings, as summarized in Table 1.

**Table 1:** Subsets and effectors of Breg

Subsets	Mouse	Human	Effector	Different mechanisms should be seperated by ENTER or semicolon.	Reference
Transitional 2 marginal zone precursor B cells/marginal zone precursor B cells	CD21^hi^CD23^+^CD24^hi^IgM^hi^IgD^+^CD1d^+^	CD23^+^sIgM^hi^sIgD^+^CD21/CD35^hi^	IL-10	Suppress Th1/17 and T-cell proliferation (add ENTER or ";")Induce Tr1/Treg cells	50,84,104,105
Marginal zone B cells	CD19^+^CD23^-^CD21^+^	–	IL-10	Suppress antigen-specific CD8^+^ T-cell and CD4^+^ T-cell response add ENTER or ";"Induce Tr1 cells	20,31,40
Peritoneal B1a B cells	CD19^+^CD5^+^CD11b^+^	–	IL-10	Regulate neutrophil infiltration (add ENTER or ";") Suppress CD4+ T-cell activation and pro-inflammatory cytokine production	20,51
Plasma cells/plasmablasts	CD138^+/hi^CD22^-^	CD27^int^CD38^+^	IL-35, IL-10, Blimp1, IRF4	Inhibit Th1/17, neutrophils, natural killer cells and dendritic cells (add ENTER or ";") Regulate B cells antigen-presenting ability and pro-inflammatory cytokine secretion (add ENTER or ";")Induce IL-10-producing B cells and IL-35-producing B cells	22,37,46
B10	CD5^+^CD1d^hi^	CD5^+^CD1d^hi^, CD24^hi^CD27^+^	IL-10	Suppress CD4^+^ T cells proliferation and pro-inflammatory cytokine secretion (add ENTER or ";") Suppress antigen-presenting function of dendritic cells Regulate monocyte cytokine production	16,31,39,52,54,55
TIM-1^+^ B cells	CD19^+^TIM-1^+^	CD19^+^TIM-1^+^	IL-4, IL-10	Promote Th2 response (add ENTER or ";") Suppress Th1/17(add ENTER or ";") Suppress antibodies secreted by plasma cells	18,21,60,61,106
Naive B cells/transitional B cells	–	CD19^+^CD24^+/hi^CD38^hi^	IL-10, TGF-β	Suppress IFN-α production by plasmacytoid dendritic cellsadd (ENTER or ";") Suppress T-cell proliferation, inhibit Th1/17 Induce Tregs and Tr1 cells	24,34,56,58,90,91,107
Circulating B cells	–	CD19^+^CD25^hi^ CD27^hi^CD1d^hi^ CD86^hi^	IL-10, TGF-β	Suppress T-cell proliferation (add ENTER or ";") Induce Tregs	68

## Identifying specific markers for Bregs

Generally, any subset of B cells that exerts immune regulatory functions can be called Bregs because there is no consensus on its definition and classification. Based on this generalized conception, Bregs consist of a diversity of subsets as shown in Table 1. Given that interleukin (IL)-10 plays a central role in immune regulation, IL-10-producing B cells are sometimes narrowly defined as “Bregs” from a functional perspective^13–15^. Meanwhile, Tedder et al. also defined a distinct subset of B cells as “B10 cells”, which reflected their unique functional program (IL-10-competent in vivo and expressing IL-10 after a 5-h stimulation with phorbol 12-myristate 13-acetate (PMA) and ionomycin *ex vivo*) and the fact that their anti-inflammatory effects were solely attributable to IL-10 production^16,17^. However, Bregs also function via IL-10-independent mechanisms including cytokines like IL-35 and transforming growth factor (TGF)-β, and direct cell–cell contact; therefore, IL-10-producing B cells are not equivalent to Bregs.

Great efforts have been conducted to identify a specific marker for Bregs, just like CD25 and Foxp3 in regulatory T cells (Tregs); however, until now, none of them has been convincing^7^. CD5^+^CD1d^hi^ B cells and T-cell Ig and mucin domain protein 1 (TIM-1^+^) B cells are promising hotspots of Bregs due to their simple cell surface markers and enrichment of IL-10-producing B cells^16–18^. As many as 15–20% of CD5^+^CD1d^hi^ B cells are B10 cells. By contrast, the percentage of B10 cells in whole splenic B cells is only 1–3%^16,17,19^. However, it must be noted that CD5^+^CD1d^hi^ B cells themselves only account for approximately 2% of whole splenic B cells; therefore, the majority of B10 cells are still within CD1d^lo^ and CD5^−^ populations^16,18^. Meanwhile, when CD5^+^CD1d^hi^ and CD5^−^CD1d^hi^ marginal zone B cells are cocultured with apoptotic cells respectively, the secretion of IL-10 increases in both groups, and the level of IL-10 has no significant difference^20^. TIM-1^+^ B cells appear to better encompass IL-10-producing B cells because nearly 70% of IL-10-producing B cells express TIM-1^18,21^. Moreover, within the subsets of the marginal zone and the transitional 2 marginal zone, as well as of follicular, CD5^+^CD1d^hi^, B1 or type 1 transitional B cells, a majority of IL-10-producing B cells are located in the TIM-1^+^ subsets rather than the TIM-1^−^ subsets. Nevertheless, expression of IL-10 and the cell surface marker TIM-1 is still not absolutely synchronous, which means it is not a specific marker for IL-10-producing B cells, let alone for Bregs.

The heterogeneity of Bregs subsets raises a critical question: are Bregs a distinct lineage where a specific factor controls the expression of genes responsible for their suppressive nature, or are they just the response of B cells to certain stimuli in a specific environment?^2,14^ The definition of B10 cells appears to support the former viewpoint because PMA and ionomycin are not able to initiate gene expression without the existence of appropriate transcription factors. Meanwhile, the majority of B cells cannot express IL-10 after the same stimulation^17^. However, these “existed factors” in B10 cells have not been identified, and the gene arrays of Bregs from mice and humans found no conclusive lineage-specific marker^14,22,23^. In addition, B10 cells require the stimulation of antigens or an inflammation environment, comparable to other Bregs subsets. The facts that immature B cells, mature B cells, and plasmablasts, as well as plasma cells could all function as Bregs^14,24–26^ and that B10 cells could inversely differentiate into antibody-secreting cells after terminating IL-10 production in vivo and in vitro^27^, further support the viewpoint that Bregs are not a distinct subset but the response of B cells to a specific environment, especially to inflammation.

## Mechanisms for Bregs induction

**Fig. 1:**
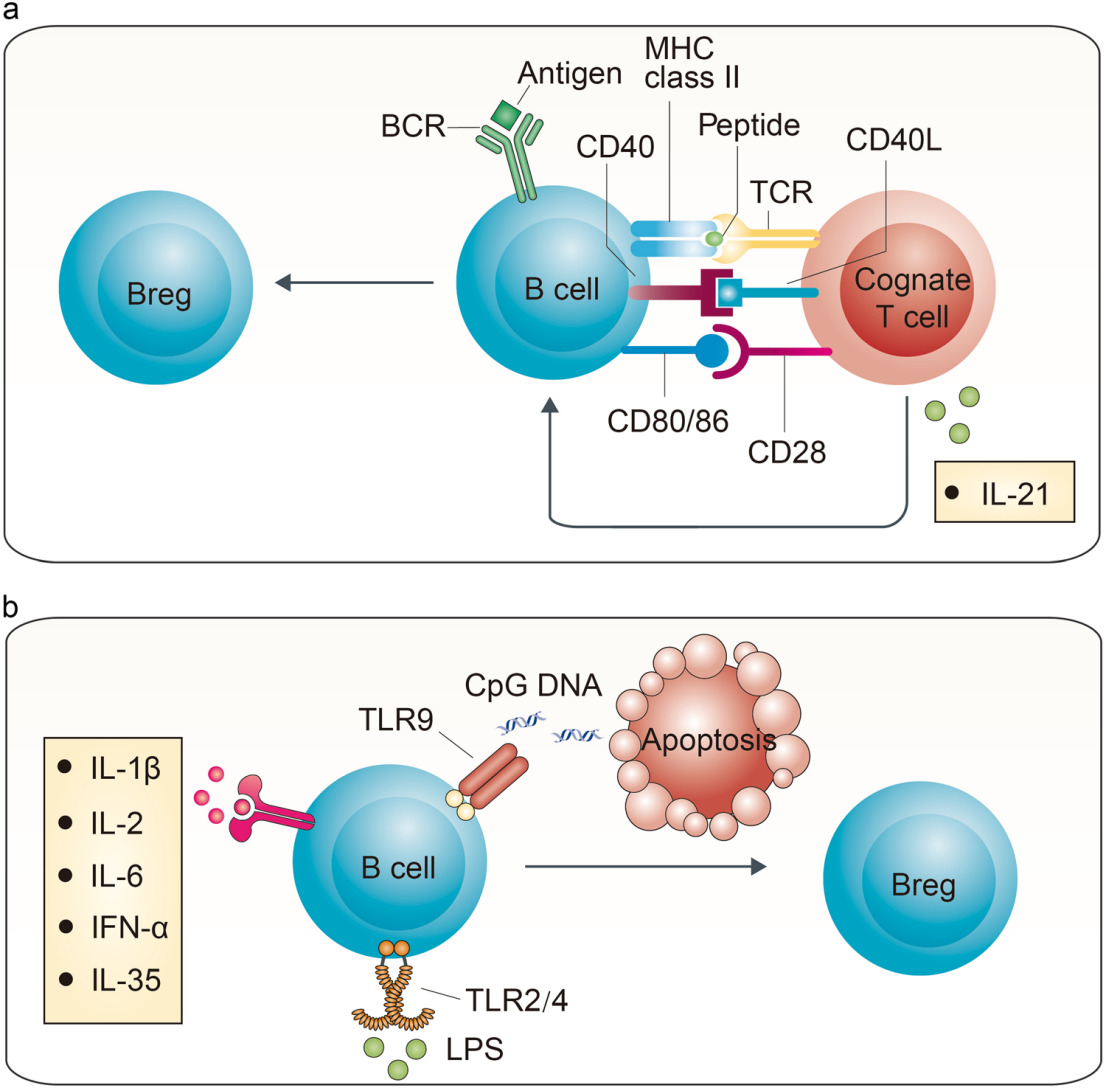
Bregs are induced through both “adaptive” and “innate” signals **a** B cells bind antigens through BCR, present them to cognate CD4^+^ T cells, become stimulated with CD40-CD40L, CD80/86-CD28, and IL-21, and then develop into Bregs. **b** LPS on TLR-2/4 and the DNA complex on TLR-9 can induce Bregs through the Myd88 pathway. Pro-inflammatory cytokines, including IL-1β, IL-2, IL-6, and IFN-α, help to induce Bregs. In addition, anti-inflammatory cytokine IL-35 also plays a role in Bregs induction through IL-12Rβ2 and IL-27Rα

If the hypothesis that Bregs acquire their regulatory ability in response to their environment is true, it is more important to determine the key mechanism that drives B cells to Bregs. Until now, some pathways have been confirmed to be necessary for Bregs induction in different settings (Fig. 1), but the entire scenario still remains mysterious.

### Pro-inflammatory signals

Inflammation is regarded as the primary requisite^14,28^. In normal states, Bregs may remain at relatively low levels to maintain immunological homeostasis. By contrast, in response to inflammation, the amount of Bregs increases, and they acquire the ability to regulate immunity^16,19^. Antigen-B-cell receptor (BCR) is believed to be involved in one of the pathways for Bregs to detect an inflammatory signal and to elicit regulatory effects. In CD19-deficient mice (deficient in BCR signal transduction), inflammation worsens, whereas in mice with CD19 overexpression, inflammation is reduced^16,29^. In addition, the absence of CD22 increases the amount of Bregs by enhancing BCR signaling^30^. Moreover, BCR makes Bregs antigen specific^20,31–33^. In TgV_H_3B4 transgenic mice, whose V_H_ is derived from an actin-reactive natural antibody, only immunization with actin significantly increases B10 cell quantities^33^. Meanwhile, after processing antigens, Bregs present them to cognate CD4^+^ T cells through major histocompatibility complex class II molecules and become stimulated via CD40L-CD40 and CD80/86-CD28 signaling pathways together with IL-21^34–36^. This indicates the importance of BCR signals and the interaction with cognate T cells for Bregs induction.

Another pathway involved in inflammation is the Toll-like receptor (TLR) for “innate type” signals^37^. The Gram-negative bacteria *Salmonella typhimurium* was found to directly induce IL-10 production by B cells in vitro via TLR-2/4 and the myeloid differentiation primary response gene 88 (Myd88) pathway^37^. Through TLR4, lipopolysaccharides (LPSs) stimulate splenic B cells to express a high level of FasL and TGF-β compared with the control^38^. In addition, LPS is also the routine constituent, together with PMA, ionomycin, and monensin, to induce IL-10 production in B10 cells^16,19,39^. TLR-9 is another receptor for detecting DNA-containing complexes on the surface of apoptotic cells, and it induces secretion of IL-10 in Bregs via MyD88 signaling^20,40–42^. The CpG oligodeoxynucleotide, which is abundant in microbial genomes and activates TLR-9, was also found to induce IL-10-producing B cells^25,43^.

In addition, other pro-inflammatory stimuli were reported. A proliferation-inducing ligand was found to induce IL-10-producing B cells through the transmembrane activator and calcium modulator and cyclophilin ligand interactor (TACI) and the downstream signal transducer and activator of transcription 3 (STAT3) pathway^44^. Plasmacytoid dendritic cells were reported to drive CD19^+^CD24^hi^CD38^hi^ immature B cells into IL-10-producing Bregs via the release of interferon-α (IFN-α) and CD40 engagement^24^. In the arthritis mice, IL-1β and IL-6 induced Bregs differentiation and IL-10 production; by contrast, in mice lacking IL-6 receptor or IL-1β receptor specifically on B cells, Bregs were reduced^45^. IL-2 might also play a role to induce CD27^int^CD38^+^ plasmablasts that predominantly secreted IL-10, together with IL-6, IFN-α, and CpG^46^.

### Anti-inflammatory signals

IL-35 is the only anti-inflammatory effector in the IL-12 family, which is critical for the regulation of T-cell-mediated autoimmunity^22^. Interestingly, this anti-inflammatory cytokine has also been shown to induce Bregs^47^. Wang et al. reported that recombinant IL-35 (rIL-35) could induce both IL-35-producing Bregs and IL-10-producing Bregs. With the stimulation of LPS and rIL-35, the IL-35-expressing population increased from 7.8 to 35.3%. Meanwhile, 17.8% of IL-35-producing Bregs co-expressed IL-10, whereas 53.6% of IL-10-producing Bregs also expressed IL-35. Further research on the mechanism of rIL-35 revealed that it functioned through the receptors of IL-12Rβ2 and IL-27Rα subunits on B cells and signaled through STAT1 and STAT3 but not STAT4 pathways^47^. These findings indicate that the induction of Bregs is not limited solely to inflammation.

## Bregs functions and effectors

**Fig. 2:**
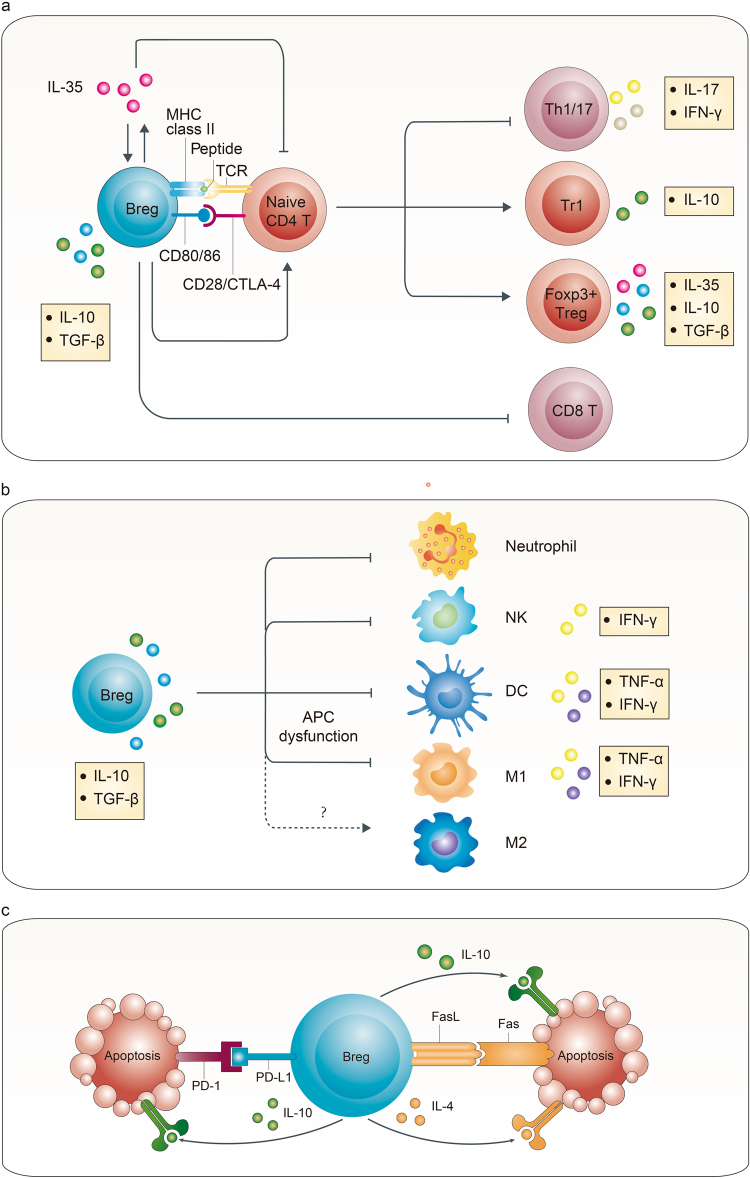
Multiple mechanisms are involved in Bregs functions **a** Through cell contact and anti-inflammatory cytokines, including IL-10, TGF-β, and IL-35, Bregs suppress CD4^+^ T-cell proliferation, induce Foxp3^+^ Tregs and Tr1 cells, and suppress Th1/17 differentiation and CD8^+^ effector T cells. **b** IL-10 and TGF-β secreted by Bregs hamper the antigen-presenting function and cytokine secretion of dendritic cells and M1 macrophages, and they possibly induce M2 macrophages. They also negatively regulate neutrophils and NK cells. **c** PD-L1 and FasL expressed on the surface of Bregs induce T-cell apoptosis through binding to PD-1 and Fas, respectively, together with cytokines IL-10 and IL-4

The central role of Bregs is to negatively regulate the immune system and help maintain immunological homeostasis. As shown in Fig. 2, multiple mechanisms are involved, including skewing T-cell differentiation, induction, and maintenance of Tregs, as well as suppression of pro-inflammatory cells^2,14,17,28,48^. Rosser et al. summarized the mechanisms into four patterns: cognate suppression, cell contact-mediated suppression, bystander suppression, and indirect suppression, which revealed the multiplicity and complexity of Bregs’ functions^49^. Based on the effectors, these functions can be divided into IL-10-dependent and -independent mechanisms.

### IL-10-dependent mechanism

IL-10 is considered the master negative regulator of inflammation and the key contributor of Bregs. IL-10 produced by CD5^+^CD1d^hi^ B cells has been found to suppress T-cell-mediated inflammation. Adoptively transferring CD5^+^CD1d^hi^ B cells from *Il10*^*-/-*^ mice did not relieve inflammation, whereas those from the wild type did^16^. Mice selectively deficient in IL-10 expression of B cells (*Il-10*^*-/-*^ B cell) could not recover from EAE, and the transferring of IL-10-producing B cells ameliorated EAE via suppression of the Th1 response^36^. Meanwhile, Carter et al. reported that *Il-10*^*-/-*^ B-cell chimeric mice showed a significant decrease in Foxp3^+^ Tregs, and *Il-10*^*+/+*^ B cells induced more Foxp3^+^ Tregs than *Il-10*^*-/-*^ B cells in response to antigen stimulation in vitro, which indicated that IL-10-producing B cells could induce Foxp3^+^ Tregs^50^. Moreover, neutrophils, natural killer cells, and dendritic cells were also reported as target cells regulated by B-cell-derived IL-10^37,46,51–53^. In humans, impairment of IL-10 production in Bregs could contribute to autoimmune diseases. The amounts of IL-10^+^CD19^+^ and IL-10^+^CD5^+^CD1d^+^ B cells, as well as the serum IL-10 concentration were significantly lower in Henoch–Schonlein purpura nephritis patients compared with healthy controls (HC); after treatment, they significantly increased. Meanwhile, the estimated glomerular filtration rate was also positively correlated with the amount of Bregs and the serum IL-10 concentration^54^. Although markers of Bregs might differ, a similar decrease or impairment of IL-10-producing B cells was also observed in patients with rheumatoid arthritis^44,55–57^, systemic lupus erythematosus (SLE)^24,55,58,59^, multiple sclerosis^26,55^, systemic sclerosis^60^, Myasthenia Gravis^61^, and other autoimmune diseases. However, interestingly, only certain mechanisms of IL-10 function in a particular circumstance. This may lead to paradoxical results in different studies.

### IL-10-independent mechanism

In addition to IL-10, IL-10-independent mechanisms have been well documented by recent studies^4,13,62^. IL-35 not only induces Bregs but also serves as an important effector of Bregs, which has a strong immunosuppressive effect^47^. In vitro, IL-35 inhibits the proliferation of B cells and T cells. In vivo, IL-35 treatment or adoptively transferring IL-35-induced Bregs ameliorates experimental autoimmune uveitis by inhibiting Th1/17 while inducing Tregs^47^. Similar results were confirmed in the model of EAE and *Salmonella enterica* infection^22^. These findings indicated that IL-35 played a unique role in Bregs effects, which contributed to forming a positive feedback network to downregulate the immune response.

TGF-β is another large family that suppresses inflammation over a wide range^63^. Tian et al. reported in 2001 that splenic B cells activated by LPS expressed high levels of TGF-β and FasL^38^. In vitro, these activated B cells inhibited the Th1 response, triggered apoptosis of splenic mononuclear cells, and impaired the function of antigen-presenting cells. In vivo, transfusion of activated B cells, rather than the control, inhibited spontaneous autoimmune diabetes in nonobese diabetic mice. Subsequently, a series of studies further demonstrated the individual function of TGF-β secreted by Bregs^64–66^. Among them, Bjarnadottir et al. reported that mice selectively deficient in TGF-β1 expression in B cells developed an earlier onset of EAE and presented with more severe central nervous system inflammation. This was related to augmented Th1/17 responses and increased myeloid dendritic cells^64^. In addition, TGF-β is known to induce Tregs. Human peripheral B cells activated by CpG could induce CD25^+^Foxp3^+^ Tregs through IL-10, indoleamine 2,3-dioxygenase enzyme and TGF-β. Blocking experiments with monoclonal antibodies (mAbs) indicated that IL-10 and TGF-β had independent and complementary roles, respectively^67^. CD25^hi^CD27^hi^CD1d^hi^CD86^hi^ Bregs in humans secreted both TGF-β and IL-10; however, the induction of Tregs and their expression of Foxp3 and CTLA-4 were dependent on TGF-β and cell–cell contact (perhaps via the CD86–CTLA-4 interaction)^68^.

The function of FasL and PD-L1 expressed on the surface of Bregs, which induces apoptosis through binding Fas and PD-1, respectively, on the target cells, has also been demonstrated^69–72^. These findings further explain the cell–cell contact dependent mechanism of Bregs.

## Pan-B-cell depletion accelerates rejection in kidney transplantation

From the conventional perspective, B cells contribute to both acute and CR by presenting antigens to T cells and by producing cytokines and alloreactive antibodies^8^. Donor-specific antibodies (DSA) produced by B cells bind to mismatched human leukocyte antigens (HLAs) or non-HLA molecules on the graft, initiate a set of signal events through complement-dependent and -independent pathways, recruit effector cells and finally lead to AMR. It is well recognized that AMR is the major cause of long-term graft loss. Therefore, B-cell research is focusing on avoiding the production of DSA and preventing AMR^3^. The pan-B-cell depletion strategy, which targets either B cells or plasma cells without discriminating effector or Bregs, including using anti-CD20 mAbs (rituximab and ofatumumab), anti-CD19 mAbs, anti-CD52 mAbs (alemtuzumab), proteasome inhibitor (bortezomib), and anti-thymocyte globulin, is believed to be useful for AMR treatment^73–82^.

However, this pan-B-cell depletion strategy does not work all of the time. Recently, a randomized, controlled trial comparing rituximab and daclizumab (anti-CD25 mAb) induction therapies in patients undergoing kidney transplantation was suspended because of a higher incidence of acute rejection in the rituximab group^83^. Both groups received two doses of induction at day 0 and day 7 (daclizumab, 1 mg/kg; rituximab, 10 mg/kg, together with 10 mg/kg methylprednisolone before rituximab) and underwent corticosteroid-free maintenance with tacrolimus and mycophenolate mofetil. Although peripheral B cells were undetectable in all patients receiving rituximab, five of six patients had biopsy-proven acute cellular rejection in the first 3 months after transplantation, whereas only one of seven patients did in the daclizumab group (83% vs. 14%, *P* = 0.01). This revealed the existence of Bregs and their immune regulatory functions in kidney transplantation and highlighted the importance to discriminate Bregs from inflammatory B cells.

In addition, similar results were observed in transplantation models. Ding et al. reported that in the model of islet transplantation, depletion of B cells prior to transplantation with anti-CD20 mAbs slightly shortened allograft survival compared with B-cell-intact untreated mice (median survival time, MST, 10 days vs. 13 days). Surprisingly, anti-TIM-1 treatment accelerated allograft rejection in B-cell depletion recipients, whereas in B-cell-intact mice, this treatment significantly prolonged allograft survival through induction of Bregs (MST, 6 days vs. 28 days)^18^. In the costimulatory blockade-induced transplantation tolerance model, depletion of B cells 1 day after cardiac transplantation prevented tolerance and led to acute cellular rejection^84^. Anti-CD20 mAb treatment also enhanced allospecific T-cell response and accelerated skin allograft rejection^85,86^. Therefore, the effect of pan-B-cell depletion may depend on the timing of B-cell depletion. When the depletion is performed during the induction period, Bregs are eliminated, leading to an accelerated T-cell response and excess allograft rejection. By contrast, when performed in an AMR setting, it mainly affects effector B cells and may help attenuate rejection^3,73^.

## Bregs contribute to tolerance in kidney transplantation

Further evidence regarding the critical role of Bregs in graft tolerance has been found in operationally tolerant (OT) patients. Although it is rare, a small group of kidney transplant individuals managed to maintain normal graft function after completely stopping immunosuppression. A widely recognized definition of operational tolerance is stable graft function without clinical features of CR in the absence of any immunosuppressive drugs for >1 year^87^. Analysis of peripheral blood cell phenotypes showed that OT patients were characterized by a higher absolute number and frequency of B cells compared with stable patients under immunosuppression (SI) and CR patients, and OT patients were comparable with the HC^88,89^. The main increase in B cells remained in the IgD^-^CD27^+^CD38^+/-^ memory subset, as well as the IgD^+^CD38^+^ subset, and expression of CD80/86, CD40, CD62L, CD5, and CD1d increased compared with stable and CR patients^88^. More importantly, these B cells displayed inhibitory profiles, including a decrease in the FcγRIIA/FcγRIIB ratio, an increase in the B-cell scaffold protein with Ankyrin repeats 1 (BANK1), which negatively regulated CD40-mediated AKT activation, and an increase in TACI vs. the B lymphocyte-stimulating factor (BAFF) ratio^88^. These special characteristics prevented hyperactive B-cell response and skewed B cells to the regulatory side.

Concurrently, similar studies were performed by the immune tolerance network (ITN) and the indices of tolerance (IOT) to identify specific biomarkers of OT patients^90,91^. The ITN study recruited a cohort of 25 OT patients and showed that only the B-cell signature was significantly different between OT and SI groups. The same observation was seen between OT and HC groups. More specifically, OT patients had increased amounts of total B cells and naive B cells (CD27^−^IgD^+^IgM^+^) compared with SI patients^90^. When the researchers of ITN combined their patients with those from the IOT study and tested more surface markers, they found an increase in transitional B cells (CD38^+^CD24^+^IgD^+^), as well as total B cells and naive B cells in OT patients compared with the SI group^90^. The same result was confirmed in their recent study^92^. In addition, these transitional B cells expressed more IL-10 in the OT group relative to the SI or HC groups^90^. The increased amount of peripheral B cells in OT patients was also observed in the study performed by IOT; however, this was observed jointly with increased NK lymphocytes and decreased activated CD4^+^ T cells^91^. Analysis of B-cell subsets also showed an increase in transitional B cells and naive B cells with a memory pool decrease. The cytokine that mediated immune regulation was possibly TGF-β rather than IL-10^91^. Nevertheless, these studies revealed that increased B cells possessing immune regulatory capability contributed to the operational tolerance of kidney transplantation.

In addition to lymphocyte phenotypes, gene expression profiles of OT patients were analyzed, and they showed a close relationship with B cells. The ITN study reported that 22 of the 30 genes that had a twofold increase in OT vs. SI tested by microarrays were B-cell specific and were mainly involved in B-cell activation and differentiation. Next, when tested with the multiplex real-time polymerase chain reaction (PCR), 31 of 228 genes were significantly different between the OT and SI groups (no difference between the OT and HC groups), and 26 of them were B-cell specific, which encoded κ/λ light chains of Ig. After linear discriminate analysis, *IGKV4-1*, *IGLL1*, and *IGKV1D-13* were most predictive of tolerance, and these genes were related to transition, class switch, and receptor editing of B cells^90^. When six newly recruited OT patients were added in their recent study and genes were re-examined together with previous patients, *IGLL1* and *IGKV1D-13* were still significantly different between the OT and SI groups, and they remained relatively stable over time in OT patients. Particularly, the single most predictive gene *IGKV1D-13* remained significantly different after correcting for total B cells; however, this was not observed for naive or transitional B-cell amounts, which suggested that naive and transitional B cells contributed to some portion of the alteration^92^. In the latest research using the expression of these two genes to predict tolerance in renal transplant patients, individuals consistently predicted to be tolerant had improved renal function, suggesting the potential protective function of Bregs^93^.

Essentially, B-cell subsets in human peripheral blood, including transitional B cells, memory B cells, and naive B cells, not only express IL-10 but also express pro-inflammatory cytokines like tumor necrosis factor-α (TNF-α) simultaneously. Among them, transitional B cells had the highest ratio of IL-10/TNF-α, which makes it the Bregs representative^94^. Interestingly, transitional B cells in kidney rejection patients expressed similar levels of IL-10 compared with those in stable patients; however, levels of TNF-α were much higher, which indicated that the immune regulatory function of transitional B cells should be characterized by the IL-10/TNF-α ratio. Moreover, the low IL-10/TNF-α ratio of transitional B cells was associated with poorer graft outcomes in a 3-year follow-up of patients with graft dysfunction^94^. A more recent study by the same group used the ratio of type 1 transitional B cells (T1) vs. type 2 transitional B cells (T2) alternatively because T1 expressed a higher IL-10/TNF-α ratio and it was easier to detect cell surface markers^95^. Validated in more kidney transplant patients and longer follow-up, the T1/T2 ratio served as an excellent predictor of graft dysfunction, even better than DSA^95^. In summary, these findings revealed that the characteristics and polarization profiles of Bregs in humans not only affected their immune regulatory functions but could also be an indicator for prognosis.

## What can we expect for Bregs in the future?

### Epigenetic regulation of Bregs

**Fig. 3:**
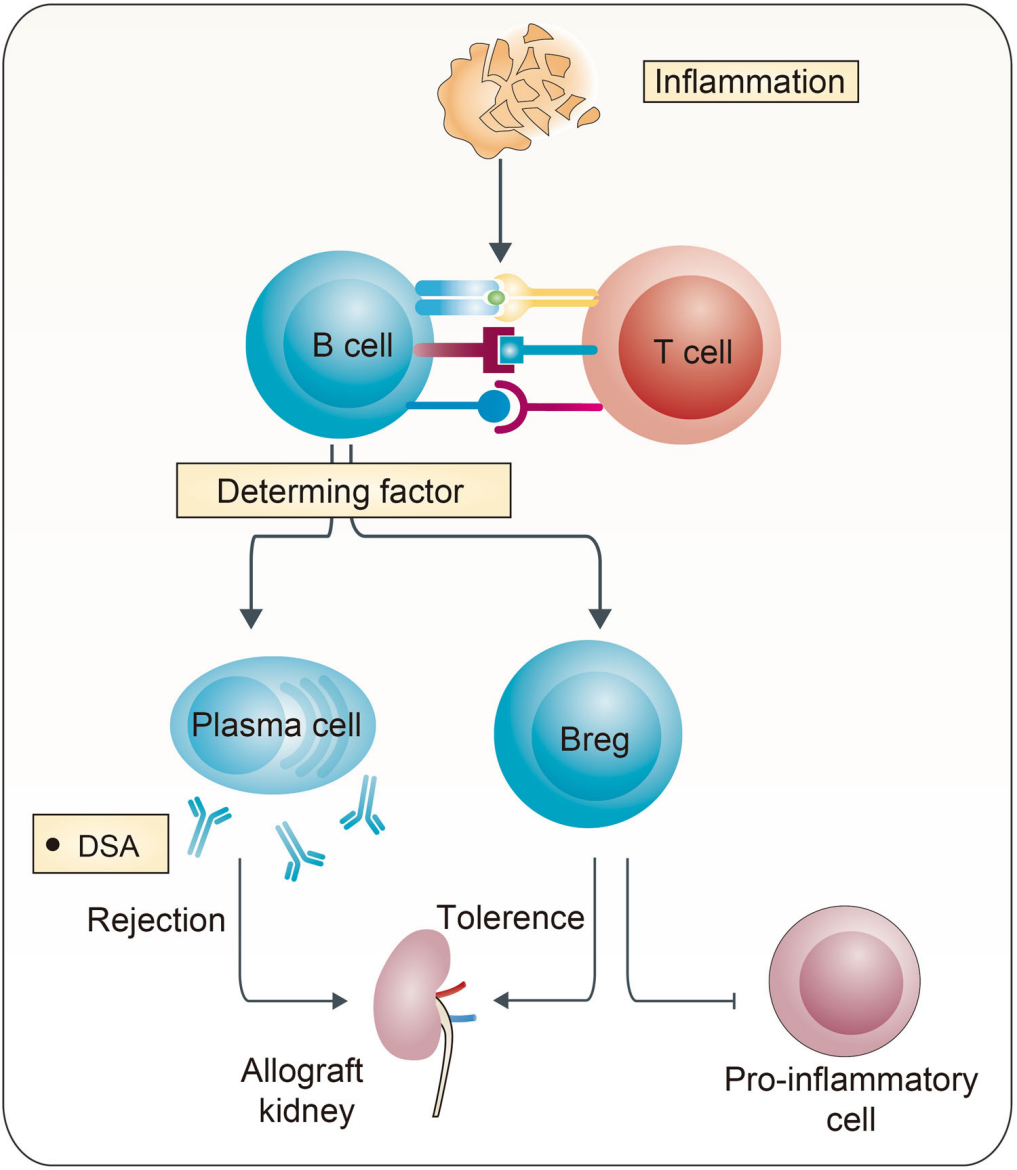
Similar environments lead to opposite differentiation of B cells B cells can develop into Bregs in response to inflammatory signals. However, similar environments can also activate B cells and stimulate them to become effector B cells. The direction of differentiation leads to a diametrically opposite consequence in kidney transplantation. Until now, determining factors still remain unknown

An abundance of research has confirmed the existence of Bregs over the last decade and highlighted their functions in tolerance induction, homeostasis maintenance, tumor metastasis, and infections. Although great progress has been achieved, the lack of the knowledge about the nature of Bregs is still an impediment to further research and applications. What specifically allows B cells to develop into Bregs? How are Bregs manipulated during development? Why could only a small population of B cells become Bregs under the same conditions? There is more to the answer than the hypothesis that Bregs are the response of B cells to inflammation (Fig. 3).

Identification of the factors that specifically make Bregs a distinct subset remains a critical and outstanding challenge. Because the surface markers are more related to the origin of B-cell subsets and the functional assay to detect intracellular cytokines (like IL-10) involves stimulation, fixation, and permeabilization, which is likely to alter the initial phenotype of B cells, transcription factors directly associated with regulatory function are suggested as better markers to identify Bregs^96^. In addition to BANK1 discussed above, IL-10-producing and IL-35-producing CD138^hi^ plasma cells were found to co-express the transcription factor B-lymphocyte-induced maturation protein 1 (Blimp1), which suppresses IL-6 secretion and distinguishes these cells from IL-10-producing CD1d^hi^ B cells^22^. Foxp3 is another well-known transcription factor that causes conventional T cells to develop into Tregs and possess suppressive activity. Recent studies revealed that Foxp3 was also expressed in the human peripheral B-cell population, which is related to milk allergy and rheumatoid arthritis^97,98^. Further research showed that LPS or IgM mAbs stimulation induced the expression of Foxp3 in more than 10% of CD19^+^ B cells, and CD19^+^Foxp3^+^ B cells suppressed proliferation of responder T cells via cell–cell contact. In vivo, adoptively transferred Foxp3-tranfected B cells inhibited autoimmune arthritis in mice through suppression of Th17 and induction of Tregs^99^. Although the origins, the surface markers or the effectors of Foxp3+ Bregs remain unclear, these results bring new insights into the future research of Bregs. Is Foxp3^+^ a common transcription factor expressed in different subsets of Bregs? How is Foxp3 expression regulated in Bregs and how does it work? Is there a specific gene region similar to the Treg-specific demethylated region in Bregs? Are methylation, acetylation, phosphorylation, or ubiquitination of certain genes and histones involved in the development and function of Bregs? Future research regarding epigenetics may help to better understand the nature of Bregs.

Additionally, microRNA (miRNA) appears to play a role in this complex network through gene expression regulation. MiR-142-3p miRNA was overexpressed in OT patients, particularly in the total B-cell population. This miRNA remained stable over time and affected nearly 1000 gene transcriptions, including key B-cell-related genes like *BANK1* and *MS4A1* (CD20)^100^. The broad range of gene networks affected was due to the incomplete pattern of miRNA target recognition. Whether this miRNA helps induce Bregs requires further research.

### Crosstalk between Bregs and other immune regulatory cells

There has been solid evidence that Bregs could induce different subsets of Tregs; however, little is known regarding their relationship with other immune regulatory cells. Myeloid-derived suppressor cells (MDSCs) are a newly identified population of regulatory cells, which are often expanded under pathologic conditions and suppress immune response^101^. Recent research reported that MDSCs could induce IL-10-producing B cells via inducible nitric oxide synthase (iNOS) in vitro and ameliorate autoimmunity in a murine model of SLE via expansion of Bregs and reduction of effector B cells^102^. However, how iNOS influenced B-cell differentiation was not elucidated. Exogenous IL-10 was reported to induce M2 macrophages in the model of myocardial infarction^103^; however, whether Bregs could induce differentiation and polarization of macrophages is unknown. Crosstalk between Bregs and regulatory dendritic cells or natural killer cells is also an interesting field awaiting exploration.

### Bregs-based cell therapy in kidney transplantation

Cell therapy is a novel promising method to promote tolerance in kidney transplantation. The ONE Study (www.onestudy.org) is a multicenter phase I/II clinical trial trying to develop immunoregulatory cell products for human organ transplantation. Currently, several trials based on Tregs are recruiting patients. Unlike Tregs, there is no clinical trial using Bregs for cell therapy, although it has been proven effective in some animal models. The main concerns lie in the lack of knowledge on Bregs induction, expansion, maintenance, and function. Before clinical application, a highly efficient induction protocol needs to be developed, and a Bregs-specific marker needs to be identified for selection. Meanwhile, it is also a critical challenge to keep Bregs stable and reduce possible risks of side effects. Therefore, there is still a long way to go before clinical application of Bregs-based therapy.

## Concluding remarks

Rapidly emerging evidence confirms the existence of Bregs and designates them as a new member of the immunosuppressive cell club. A variety of Bregs subsets have been identified, and multiple pathways and effectors are involved. Particularly in kidney transplantation, we should consider the “B side” when we are pursuing immune tolerance: on one side, B cells produce alloantibodies and lead to AMR and even graft loss; on the other side, B cells display immune regulatory functions and help induce tolerance. At present, the B-cell signature may help identify tolerant patients to minimize immunosuppressive regimens or to conduct drug withdrawals. In the future, B-cell-based cell therapy may be available for tolerance induction along with a better understanding of Bregs.
